# Nest‐Site Selection in Barn Swallows ( *Hirundo rustica* ): Patterns Consistent With the Human Proximity Hypothesis Across Three Regions of China

**DOI:** 10.1002/ece3.74071

**Published:** 2026-07-19

**Authors:** Cong Peng, Kangning Luo, Kui Yan, Wei Liang

**Affiliations:** ^1^ Ministry of Education Key Laboratory for Ecology of Tropical Islands, Key Laboratory of Tropical Animal and Plant Ecology of Hainan Province, College of Life Sciences Hainan Normal University Haikou China

**Keywords:** barn swallow, human proximity hypothesis, indoor nests, inhabited buildings, new buildings, old buildings, outdoor nests, uninhabited buildings

## Abstract

Nest‐site selection is critical to avian reproductive success. Although human activity is known to influence nest‐site choice in birds, whether humans facilitate or impede avian reproduction remains a matter of debate. The human proximity hypothesis proposes that birds breeding near human habitation experience reduced rates of nest predation and brood parasitism. To test this hypothesis, we investigated nest‐site selection in barn swallows ( *Hirundo rustica* ) across three provinces in China: Hainan, Guizhou, and Heilongjiang. Occupancy rates of the breeding swallow nests were 34.6% in inhabited buildings but fell sharply to 0.9% in uninhabited buildings. Among all active nests recorded, 98.1% were located in inhabited buildings, and swallows showed a strong preference for old buildings (91.9%). Outdoor nests (75.1%) significantly outnumbered indoor nests (24.9%), and outdoor nests were placed at significantly greater heights than indoor nests. Our findings are consistent with the human proximity hypothesis, suggesting that barn swallows maintain close associations with humans and may benefit from reduced predation and parasitism risk associated with human proximity.

## Graphical abstract

Whether humans facilitate or impede avian reproduction remains a matter of debate. Barn swallows preferentially seek human protection by nesting in inhabited buildings, show a marked preference for older structures. Swallow outdoor nests are higher than indoor nests.

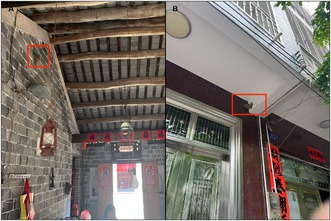

## 1 Introduction

Nest‐site selection is the process by which birds identify and occupy habitats that are most conducive to successful reproduction (Cody 1985), and nest‐site quality is a critical determinant of breeding success (Millones and Frere 2017). Natural selection favors individuals that locate safe, concealed nesting sites (Martin 1995). High‐quality sites can create locally favorable microclimatic conditions (Kesler and Haig 2005), enhance reproductive output (Martin 1995), and reduce the risk of both nest predation (Haynes et al. 2014) and brood parasitism (Liang et al. 2013; Ma et al. 2018). For instance, the superb lyrebird ( *Menura novaehollandiae* ) builds elevated nests to prevent water ingress and maintain a dry nest micro‐environment (Maisey et al. 2016). The tawny frogmouth ( *Podargus strigoides* ) orients its nest on the leeward, northeast‐facing side of trees to maximize morning sunlight exposure while providing partial wind protection, thereby optimizing nest temperature regulation (Rae and Rae 2014). The Yelkouan shearwater ( *Puffinus yelkouan* ) selects nest sites with greater concealment to minimize detection by predators and improve breeding success (Bourgeois and Vidal 2007). Laughing doves ( *Spilopelia senegalensis* ) select nest sites that are invisible from both the front and the sides, thereby reducing predation risk (Banisaffar and Shabani 2024).

Nest‐site selection is shaped by multiple interacting factors, including ecological conditions (Jovani and Tella 2004), food availability (Rather et al. 2020), nest predation (Quintana and Yorio 1998), human activity (Chen et al. 2011), access to nest materials (Hansell 2000), and brood parasitism (Feeney et al. 2012), and is therefore best understood as an integrated response to a suite of competing selective forces. Among these, avoidance of nest predation is generally considered the primary selective pressure, driving a range of behavioral and ecological adaptations. Some species exploit human presence as a form of protection. For example, the scaly‐breasted munia ( *Lonchura punctulata* ) nests in roadside vegetation along busy thoroughfares, using human traffic to deter predators (Zhou et al. 2020), while the oriental reed warbler ( *Acrocephalus orientalis* ) nests near human settlements to reduce parasitism by the common cuckoo ( *Cuculus canorus* ) (Møller et al. 2016). Additionally, the Daurian redstart ( *Phoenicurus auroreus* ) shows a strong preference for nesting inside human structures, where human presence significantly reduces predation and cuckoo parasitism rates (Zhang et al. 2023). Notably, nest abundance declines sharply following human abandonment of buildings (Zhang et al. 2020; Yao et al. 2023). Conversely, human activity can also exert negative effects on nest‐site choice, operating at different spatial scales. At the landscape scale, great cormorants ( *Phalacrocorax carbo* ) tend to select nest sites away from human settlements, effectively avoiding areas of high human activity (Guo et al. 2023). In contrast, synanthropic species such as the Eurasian magpie ( *Pica pica* ) can occupy highly urbanized environments, but within these habitats they exercise microhabitat selection: they preferentially nest in tall trees at great heights to reduce threats from humans and pets (e.g., cats) (Wang et al. 2008; Jokimäki et al. 2017; Xu et al. 2020). Thus, whether human activity exerts a net positive or negative influence on avian nest‐site selection remains an open question (Zhang et al. 2020).

The barn swallow ( *Hirundo rustica* ) breeds in the Northern Hemisphere during summer and wintering in the Southern Hemisphere (Tian et al. 2024) and has long coexisted with humans and enjoys a nearly cosmopolitan distribution (Dor et al. 2010), making it an ideal species for studying the influence of human activity on avian nest‐site selection. Research in South Korea has shown that barn swallows preferentially nest in inhabited buildings (Jeong et al. 2022; Kim et al. 2023) and that nests in areas of high human activity are associated with greater numbers of fledglings and higher overall breeding success (Kim et al. 2023). In China, a tendency to nest in human structures may be a key reason why barn swallows are rarely parasitized by cuckoos, and nesting indoors has been identified as a particularly effective defensive strategy (Liang et al. 2013). These observations are consistent with the human proximity hypothesis (Møller 2010; Liang et al. 2013), which posits that breeding near human habitation reduces the risk of both brood parasitism and nest predation (Liang et al. 2013; Møller et al. 2016). Although Zhao et al. (2022) sampled barn swallow populations across multiple latitudes in China, their study focused on life‐history traits and micro‐environmental exposure rather than on the consistency of physical nest‐site type selection (e.g., eaves vs. indoor sites). Consequently, whether the nest‐site preferences documented in South Korea—such as the preference for inhabited and old buildings—generalize across contrasting climatic and cultural settings remains unknown. By sampling across tropical, subtropical, and temperate zones in China, our study directly tests this hypothesis.

Therefore, in the present study, we aimed to examine nest‐site selection and its geographic variation across multiple barn swallow populations in China. By comparing nesting rates in inhabited versus uninhabited buildings, we investigated the role of human activity in barn swallow nest‐site choice. We also recorded building age, nest height, and nest position (indoor versus outdoor) to characterize nesting preferences. Based on the human proximity hypothesis, we made the following predictions: (1) barn swallows preferentially nest in inhabited buildings (Jeong et al. 2022; Kim et al. 2023); (2) given that indoor nesting represents a more effective anti‐predator and anti‐parasite strategy, indoor nests outnumber outdoor nests (Liang et al. 2013); and (3) because old buildings are more likely to be inhabited and provide ready‐made nesting structures, barn swallows would use old buildings more frequently than new buildings (Chen et al. 2025).

## 2 Materials and Methods

### 2.1 Study Area

**FIGURE 1:**
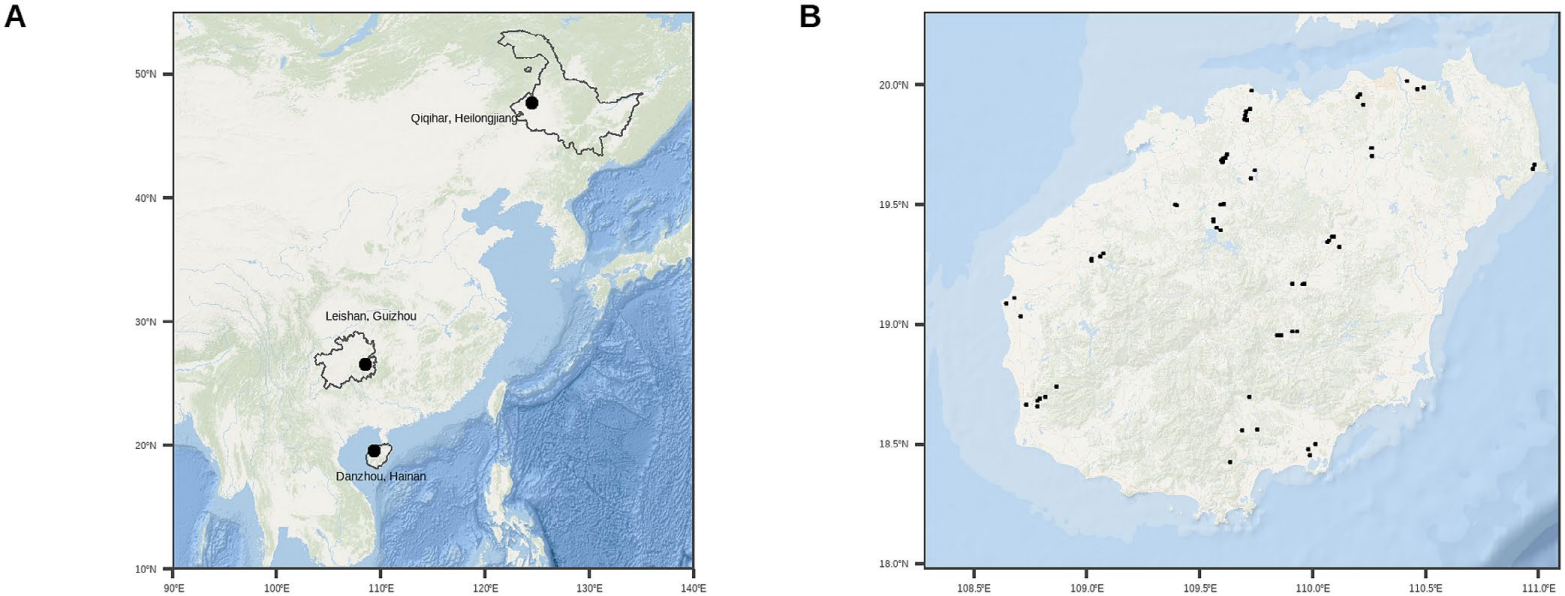
Study area. (A) Study sites for 2023–2024, comprising Danzhou City (Hainan Province), Leishan County (Guizhou Province), and Qiqihar City (Heilongjiang Province) in China. (B) Study site for 2014, comprising 69 villages across multiple municipalities of Hainan Province.

To investigate the effects of human activity on nest‐site selection in barn swallows, we selected Danzhou City in Hainan Province, Leishan County in Guizhou Province, and Qiqihar City in Heilongjiang Province as study sites (Figure 1A).

Danzhou City represents a low‐latitude tropical monsoon climate zone. Hainan Province (18°10′–20°10′ N, 108°37′–111°03′E) is located at the southernmost tip of China and has a tropical monsoon climate, with a mean annual temperature of 22°C–27°C and annual precipitation ranging from 1000 to 2600 mm. Summers are hot and rainy, whereas winters are mild and relatively dry. The province has a total land area of 35,400 km^2^ and a permanent population of approximately 10.48 million. In the 2014 breeding season, our team conducted a baseline survey of barn swallow nest sites across 11 cities and counties on Hainan Province, including Wenchang, Lingao, Changjiang, Danzhou, Dongfang, Ledong, Baoting, Lingshui, Haikou, Qiongzhong, and Tunchang (Figure 1B). Based on this survey, Danzhou City was selected as the tropical study site. Danzhou covers a total area of 3405.39 km^2^, and the habitats are dominated by tropical rainforest and mangrove wetlands. The proportion of built‐up area is 1.58%, the cultivated land area is 1338.6 km^2^, and the permanent population is about 1.09 million. The study was conducted during the 2023–2024 breeding seasons.

Leishan County represents a mid‐subtropical humid monsoon climate. Leishan County (26°02′–26°34′N, 107°55′–108°22′E) is located in the southwest of Qiandongnan Miao and Dong Autonomous Prefecture, at an altitude of 480–2178 m, with a mean annual temperature of 14°C–15°C and annual precipitation of 1310 mm. The county covers an area of 1204.36 km^2^, and the habitats are dominated by montane forests and valley farmland. The proportion of built‐up area is 0.67%, and the population is about 160,000. The study was conducted during the 2023 breeding season.

Qiqihar City in Heilongjiang Province represents a high‐latitude temperate continental monsoon climate. Located on the Songnen Plain (46°13′–48°56′N, 122°24′–126°41′E), the area has a mean annual temperature of 0.7°C–4.2°C and annual precipitation of 400–550 mm (Yan and Liang 2024). Fieldwork was carried out in Zhalong Town, Tiefeng District. Zhalong Town covers an administrative area of approximately 564.6 km^2^ and has a population of about 29,000. The built‐up area accounts for less than 0.1% of the total land, and the landscape is dominated by reed marshes and farmland. The study was conducted during the barn swallow breeding season of 2024 (Figure 1A).

### 2.2 Field Surveys

During the 2014 survey in Hainan Province, we selected 10 county‐level municipalities across the eastern, western, southern, northern, and central regions of Hainan Island, taking into account the island's topographic diversity, and sampled coastal villages, lowland foothill villages, and montane forest villages as survey units. Our study was conducted in rural village settlements across three Chinese provinces: Hainan, Guizhou, and Heilongjiang (see Study sites for detailed descriptions). The settlements were predominantly old, established villages. In some cases, residents had relocated to newly built housing, leaving former dwellings unoccupied; in other cases, entire villages had moved to new sites nearby, resulting in large numbers of abandoned buildings. Settlement density remained high in other areas. Consequently, our survey sites included a representative mix of inhabited and uninhabited buildings.

In 2014, we carried out a baseline nest census in 69 villages across Hainan. In each village, we systematically counted all buildings and classified them as inhabited or uninhabited based on daily human presence. We recorded the number of barn swallow nests in each building type (with zero recorded where none were found) and noted whether each nest was located indoors or outdoors. In total, we surveyed 4613 buildings, comprising 3695 inhabited (80.1%) and 918 uninhabited buildings (19.9%), and documented 1288 barn swallow nests.

Between 2023 and 2024, we conducted detailed nest‐site surveys in Danzhou (Hainan), Leishan (Guizhou), and Qiqihar (Heilongjiang), focusing on active nests with confirmed breeding activity. Upon locating an active nest, we recorded a series of nest‐site parameters, including nest height (the vertical distance from the nest floor to the ground), building age, occupancy status, and nest position (indoor versus outdoor). Nest height was measured as the vertical distance from the nest floor to the floor of the balcony or room where the nest was located, rather than from ground level, because ground‐level measurements would conflate nest height with building height. We note that the relationship between nest height and ceiling height differed by building type: modern houses in our study sites were typically single‐storey dwellings with similar indoor and outdoor ceiling heights, whereas traditional buildings tended to have sloping roofs, resulting in higher indoor ceilings than outdoor ceilings. Building age was categorized as “new” (≤ 5 years old) or “old” (> 5 years old) based on the report of the building's occupants. The five‐year threshold was chosen because building materials in the study regions typically undergo noticeable weathering within approximately 5 years, increasing surface roughness and providing superior attachment points for mud nests, and because a five‐year interval provides sufficient time for old nests from previous breeding seasons to accumulate (Chen et al. 2025). As building age was determined by homeowner report, it should be interpreted as an approximate estimate. At the Heilongjiang site, barn swallows commonly nested in livestock enclosures (e.g., cattle, horse, sheep, and chicken pens) and storage buildings. In this study, livestock enclosures were classified as “inhabited” only when they were physically attached to or immediately adjacent to the family dwelling and were accessed by household members multiple times daily for feeding, cleaning, or herding. This definition was based on sustained daily human presence—the ecologically relevant disturbance regime for nest‐site selection—rather than on sleeping occupancy. To ensure data consistency, all surveyors participated in a pre‐survey training session in which standardized classification criteria were discussed and practised using reference examples. Written operational definitions were provided and applied consistently across all sites and years. All classifications were made following a standardized protocol established during this training. In total, we recorded detailed nest‐site data for 942 nests: 373 in Hainan, 360 in Guizhou, and 209 in Heilongjiang.

The 2014 survey was designed as a village‐level census to document the overall distribution of barn swallow nests across Hainan. It recorded all visible nests, including old, inactive ones that may have persisted from previous breeding seasons. Two methodological differences preclude direct quantitative comparison with the 2023–2024 dataset: first, inactive and active nests were not reliably distinguished in 2014, whereas the 2023–2024 surveys targeted only active nests; second, the 2014 sampling was census‐based at the village level, whereas the 2023–2024 surveys followed a targeted active‐nest protocol. Accordingly, the 2014 data are used here solely as a qualitative baseline for inferring broad, long‐term patterns.

### 2.3 Statistical Analysis

2014 survey (Hainan). Data from all 69 villages were pooled. A 2 × 2 contingency table was constructed for building occupancy status (inhabited vs. uninhabited) and nesting status (with nests vs. without nests), and a Pearson chi‐square test of independence was used to compare nesting rates between inhabited and uninhabited buildings. For nest position, a chi‐square goodness‐of‐fit test was conducted under the null hypothesis that indoor and outdoor nests occur with equal frequency (expected ratio 1:1).

2023–2024 surveys (three regions). To test whether barn swallows exhibited an overall tendency toward particular categories of building age, occupancy status, or nest position, we pooled data from the three regions and performed one‐sample chi‐square goodness‐of‐fit tests on each binary variable, with the null hypothesis that the two categories were equally probable (50% each). To compare nest‐site selection among regions, we used Pearson chi‐square tests of independence on *R* × C contingency tables for building age, occupancy status, and nest position, respectively. For nest height, we first assessed normality (Shapiro–Wilk test) and homogeneity of variances (Levene's test). When both assumptions were met, independent‐sample *t*‐tests were used to compare nest heights between inhabited and uninhabited buildings, and between indoor and outdoor nests; when variances were unequal, Welch's corrected *t*‐test was applied; and when data deviated severely from normality, the Mann–Whitney *U* test was employed.

All statistical analyses were performed using IBM SPSS 25.0 (IBM Corp., Armonk, NY, USA), with a significance threshold of *p* < 0.05 (two‐tailed). Descriptive data for continuous variables are presented as mean ± standard deviation (SD); standard error (SE) and coefficient of variation (CV) are also reported where appropriate.

## 3 Results

### 3.1 Nest Position

**FIGURE 2:**
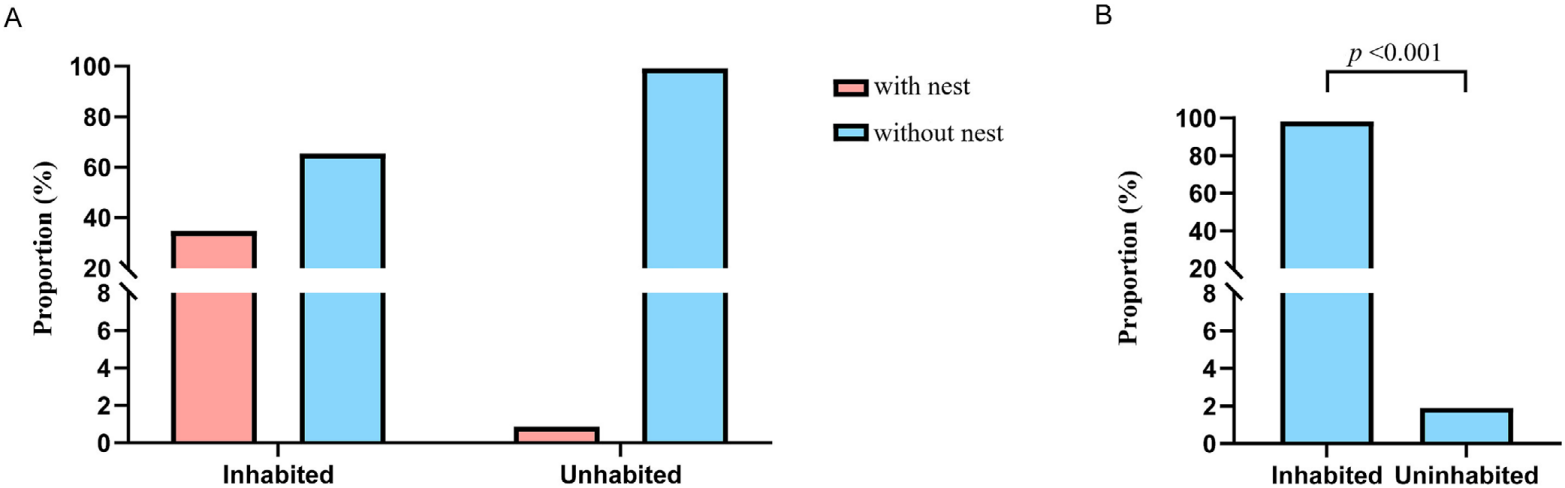
Barn swallow nesting rates in inhabited versus uninhabited buildings. (A) Nest occupancy rates in inhabited and uninhabited buildings across 69 Hainan villages in 2014. Percentages indicate the proportion of buildings with nests. Inhabited buildings: 34.6% (with nest) vs. 65.4% (without nest); uninhabited buildings: 0.9% (with nest) vs. 99.1% (without nest). *χ* ^2^ test, *p* < 0.001. (B) Comparison of the proportion of nests found in inhabited versus uninhabited buildings at the three study sites during 2023–2024.

The 2014 Hainan dataset revealed a nest occupancy rate of 34.6% in inhabited buildings (*n* = 3695) compared with a rate of 0.9% in uninhabited buildings (*n* = 918). A Chi‐square test confirmed that this difference was significant (*χ* ^2^ = 416.7, df = 1, *p* < 0.001) (Figure 2A). Similarly, analysis of the 2023–2024 data across all three sites showed that barn swallows predominantly nested in inhabited buildings (98.1%, *n* = 924) rather than uninhabited buildings (1.9%, *n* = 18), a difference that was also significant (df = 1, *χ* ^2^ = 871.4, *p* < 0.001) (Figure 2B).

**FIGURE 3:**
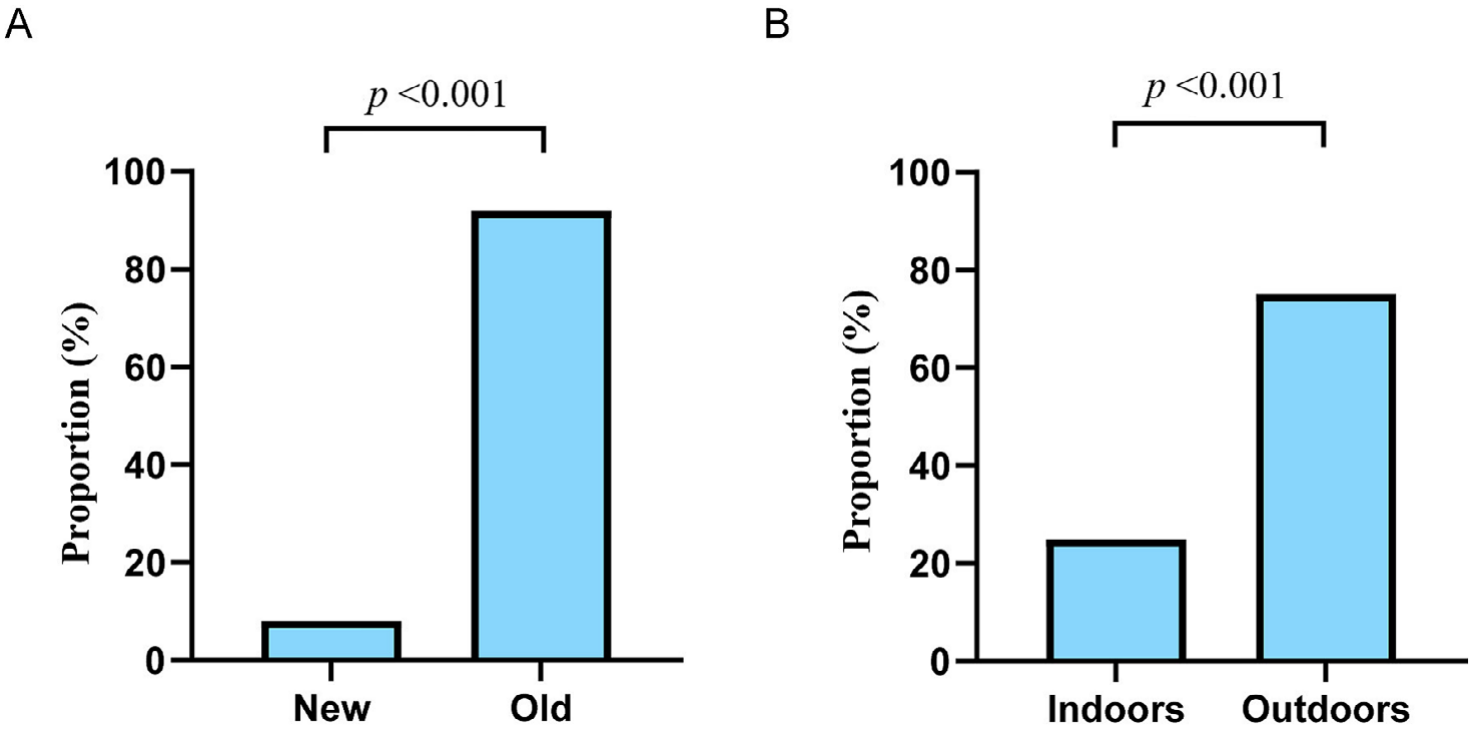
Proportions of barn swallow nests by nest‐site category across the three study sites in 2023–2024. (A) Proportion of nests in new versus old buildings. (B) Proportion of indoor versus outdoor nests.

Chi‐square analysis of the 2023–2024 data showed that barn swallows nested significantly more often in old buildings (91.9%, *n* = 866) than in new buildings (8.1%, *n* = 76) (df = 1, *χ* ^2^ = 662.5, *p* < 0.001) (Figure 3A). Furthermore, outdoor nests (75.1%, *n* = 707) were significantly more common than indoor nests (24.9%, *n* = 235) (df = 1, *χ* ^2^ = 236.5, *p* < 0.001) (Figure 3B).

**FIGURE 4:**
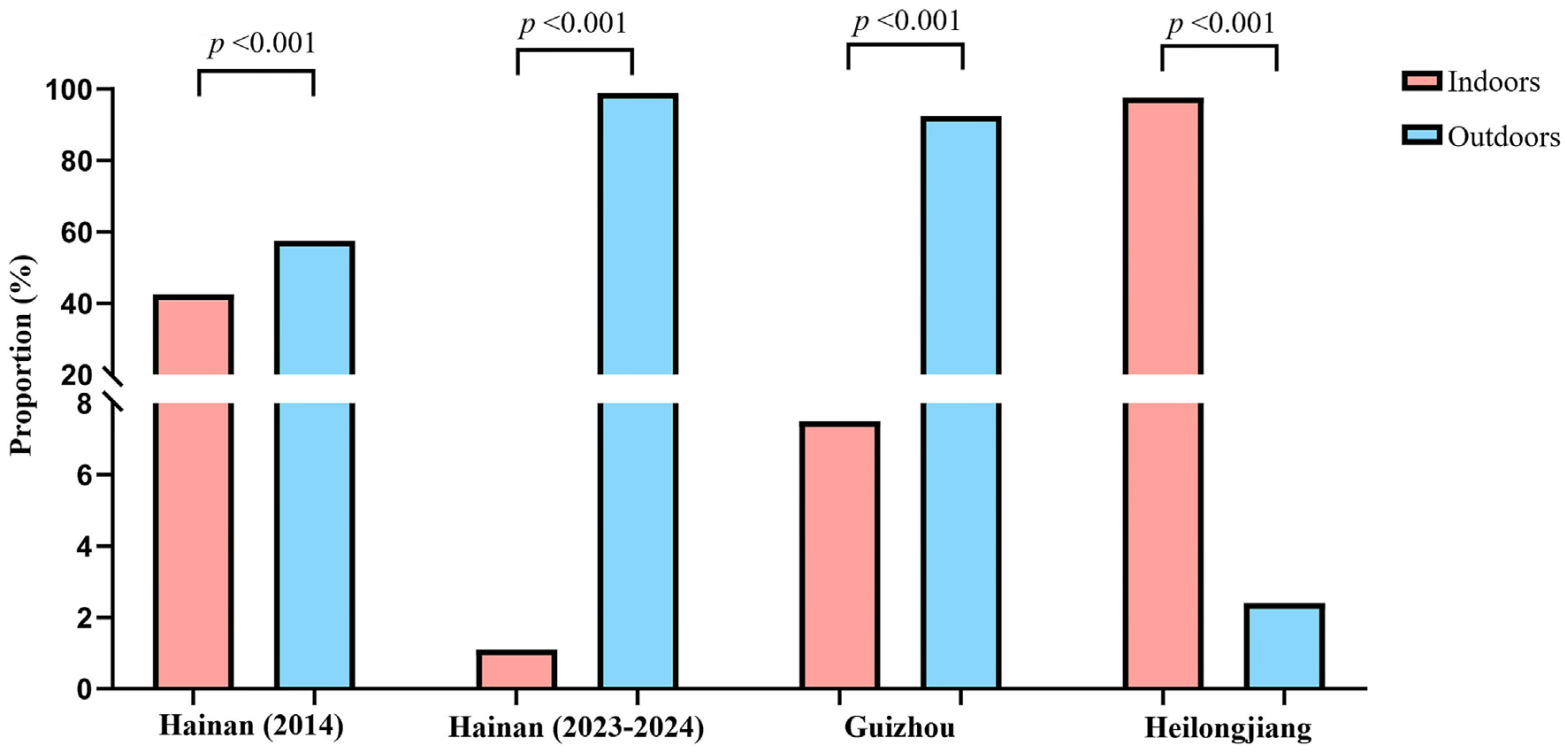
Proportion of indoor and outdoor barn swallow nests across the three study regions (2023–2024). The 2014 Hainan data are shown for qualitative reference only and are not included in statistical comparisons owing to methodological differences between the two survey periods (see Methods).

For the 2014 Hainan data, outdoor nests accounted for 57.5% (*n* = 740) of all nests across both inhabited and uninhabited buildings, significantly outnumbered indoor nests (42.5%, *n* = 548) (df = 1, *χ* ^2^ = 28.6, *p* < 0.001). In the 2023–2024 Hainan data, outdoor nests were even more predominant (98.9%, *n* = 369) compared with indoor nests (1.1%, *n* = 4) (df = 1, *χ* ^2^ = 357.2, *p* < 0.001) (Figure 4).

**FIGURE 5:**
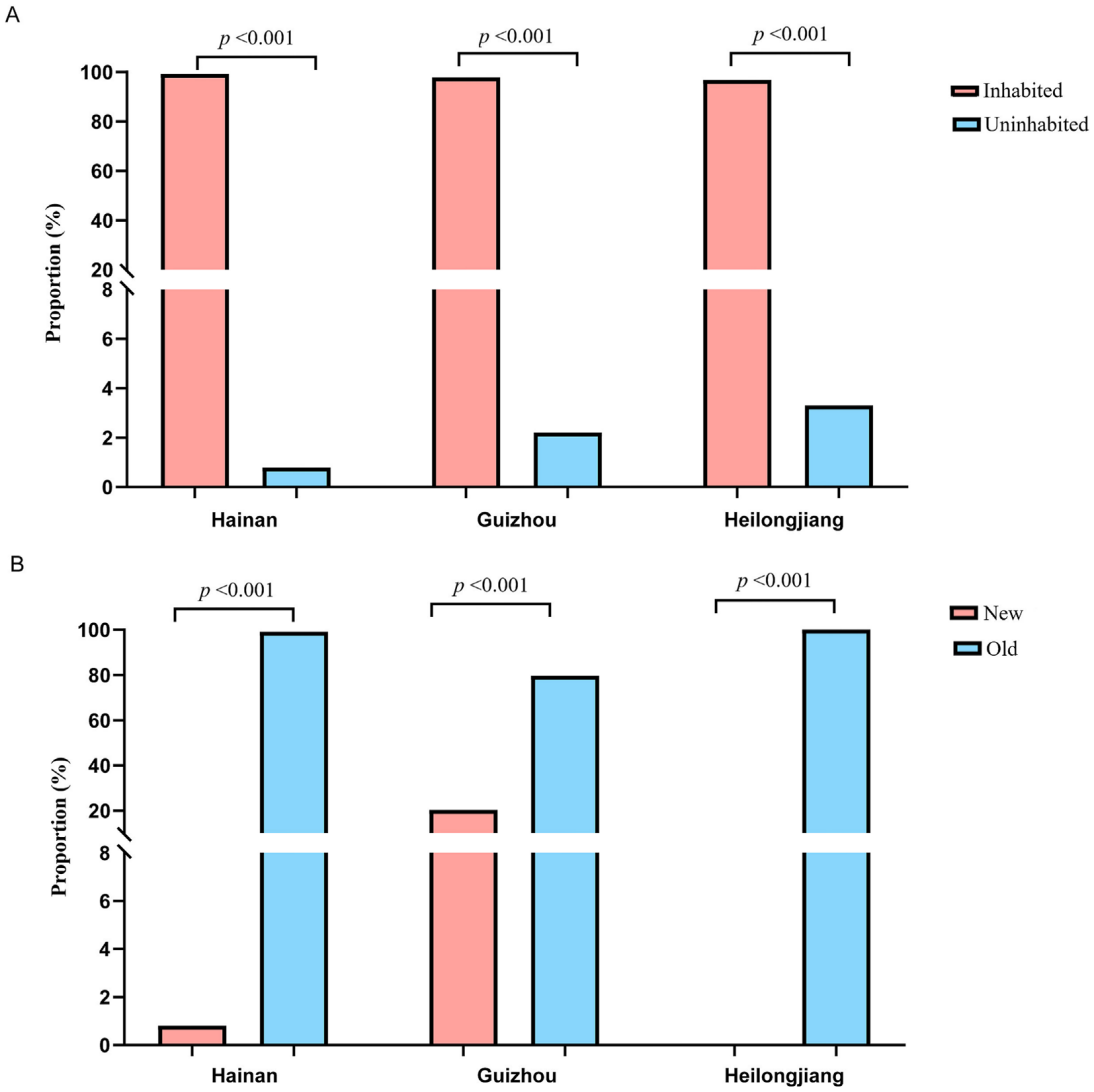
Comparison of nest‐site selection across the three study sites. (A) Proportion of nests in inhabited versus uninhabited buildings. (B) Proportion of nests in new versus old buildings.

Pairwise comparisons of the 2023–2024 data across sites revealed that nesting in inhabited buildings was the predominant pattern in all three provinces, although the proportion was significantly higher in Hainan (99.2%) than in Heilongjiang (96.7%) (*χ* ^2^ = 5.14, df = 1, *p* = 0.023) (Figure 5A). All three sites showed a majority of nests in old buildings; however, pairwise Chi‐square tests revealed significant differences between Hainan (99.2%) and Guizhou (79.7%) (*χ* ^2^ = 74.8, df = 1, *p* < 0.001) and between Guizhou and Heilongjiang (100%) (*χ* ^2^ = 48.6, df = 1, *p* < 0.001), with Hainan and Heilongjiang both having significantly higher proportions of nests in old buildings than Guizhou (Figure 5B). Regarding nest position, Heilongjiang had significantly more indoor nests (97.6%, *n* = 204) than outdoor nests (2.4%, *n* = 5) (*χ* ^2^ = 189.5, df = 1, *p* < 0.001), while Hainan and Guizhou were both dominated by outdoor nests. Pairwise comparisons of outdoor nesting proportions revealed significant differences between Hainan (98.9%) and Guizhou (92.5%) (*χ* ^2^ = 18.69, df = 1, *p* < 0.001), between Hainan (98.9%) and Heilongjiang (2.4%) (*χ* ^2^ = 543.5, df = 1, *p* < 0.001), and between Guizhou (92.5%) and Heilongjiang (2.4%) (*χ* ^2^ = 445.2, df = 1, *p* < 0.001), yielding an outdoor nesting gradient of Hainan > Guizhou > Heilongjiang (Figure 4). The proportion of outdoor nests differed significantly among the three regions, following a gradient from Hainan (highest) to Heilongjiang (lowest).

### 3.2 Nest Height

**FIGURE 6:**
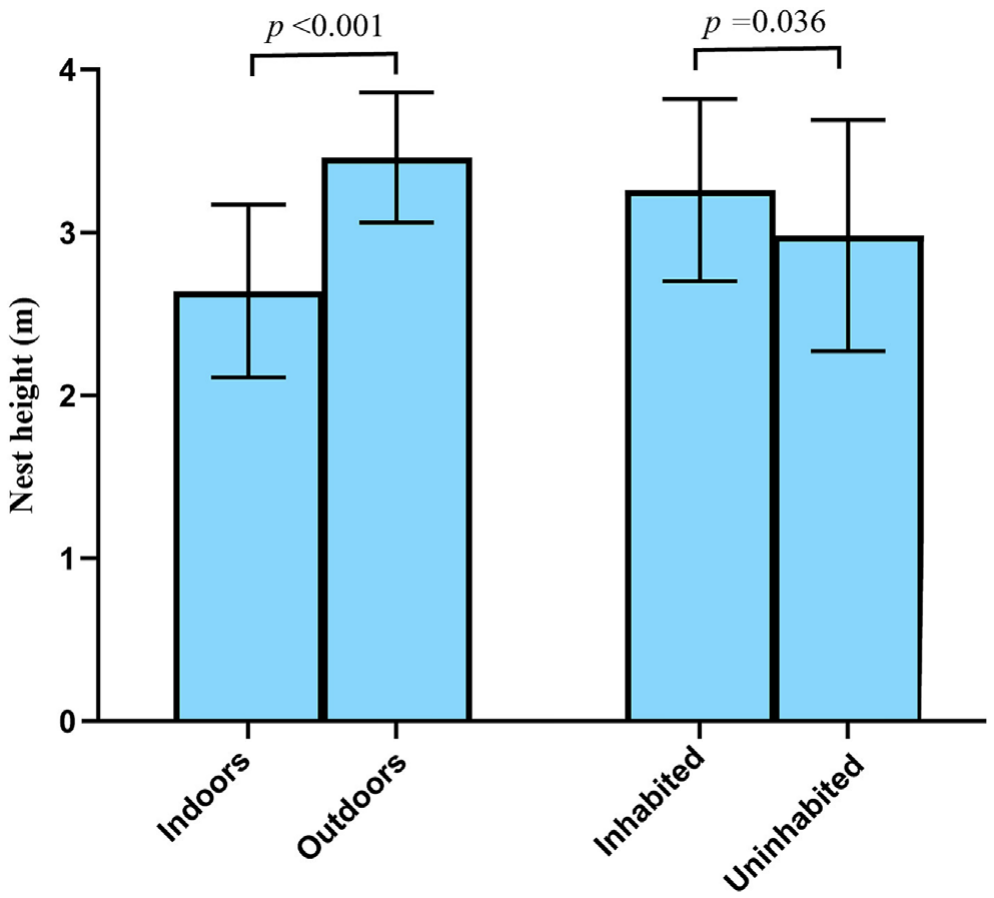
Differences in nest height between indoor and outdoor nests and between nests in inhabited and uninhabited buildings.

Mean nest height was significantly lower for indoor nests (2.64 ± 0.53 m, *n* = 235) than for outdoor nests (3.46 ± 0.40 m, *n* = 707) (df = 940, |*t*| = 21.59, *p* < 0.001). Nest in inhabited buildings (3.26 ± 0.56 m, *n* = 924) were placed at significantly higher heights than those in uninhabited buildings (2.98 ± 0.71 m, *n* = 18) (df = 940, |*t*| = 2.1, *p* = 0.036) (Figure 6). Given the highly unbalanced sample sizes, this result should be interpreted with caution.

## 4 Discussion

Our results show that barn swallows used inhabited buildings more frequently than uninhabited ones and old buildings more frequently than new ones, which is consistent with predictions (1) and (3). However, contrary to prediction (2), outdoor nests were more common than indoor nests. Qualitative observations suggest that outdoor nesting has become more prevalent in Hainan over the past decade, coinciding with rapid urbanization and architectural change, although direct quantitative comparison between periods is precluded by methodological differences (see Methods).

### 4.1 Preference for Inhabited Buildings

The higher nesting rate observed in inhabited buildings may be explained by several non‐mutually exclusive factors. First, human presence may deter nest predators such as snakes, rodents, owls, bats, and raptors (Møller 1987, 1989, 2010), and nests in areas of higher human activity have been associated with greater fledgling numbers (Kim et al. 2023). Second, inhabited buildings tend to be structurally sounder than abandoned ones, potentially offering more stable nesting substrates; in contrast, abandoned buildings are more susceptible to structural deterioration, which may reduce nest persistence (Jeong et al. 2022). Third, artificial light at night in occupied buildings may allow extended foraging and provisioning (Wang et al. 2021), a condition unavailable in uninhabited buildings, and nighttime light intensity has been positively associated with nest occurrence probability (Chen et al. 2025). Fourth, brood parasites may avoid areas of frequent human activity, potentially lowering parasitism risk (Liang et al. 2013; Møller et al. 2016). Enhanced food availability near dwellings due to livestock and organic waste could also play a role, but uninhabited buildings with similar surrounding vegetation were rarely used, and outdoor nests were concentrated near doorways regardless of proximity to livestock or light sources. These patterns are more consistent with a deterrence function of human presence than with a purely resource‐based explanation. Furthermore, the few nests in uninhabited buildings were typically situated close to regular human activity (e.g., buildings visited daily for livestock tending), reinforcing the importance of proximity to humans. Accessibility alone (open doors/windows) cannot fully account for the preference, as structurally similar but unoccupied buildings were consistently avoided.

### 4.2 Preference for Old Buildings

The tendency to use old buildings may be attributable to several factors. Old buildings often possess structural features suitable for nesting, such as rough textured surfaces, overhanging elements, and lower story heights, and building age is positively associated with nest occurrence probability (Chen et al. 2025). Old buildings are also more likely to contain existing nests from prior breeding seasons. Nest reuse is common in barn swallows, with reported rates ranging from 50% to 80% (Barclay 1988; Safran 2004, 2006; Shields 1984). Reusing an existing nest is associated with earlier breeding initiation, reduced energetic costs, and higher reproductive success (Safran 2004, 2006; Teglhøj 2018). Old nests may also function as social cues, signaling a safe site over multiple seasons (Ringhofer and Hasegawa 2014). Additionally, old buildings are often surrounded by more mature vegetation due to the “legacy effect” (Clarke et al. 2013; Li et al. 2025), which may support higher flying insect abundance and provide more reliable foraging opportunities (Navarro et al. 2026; Campera et al. 2024).

### 4.3 Why Outdoor Nests Outnumber Indoor Nests

**FIGURE 7:**
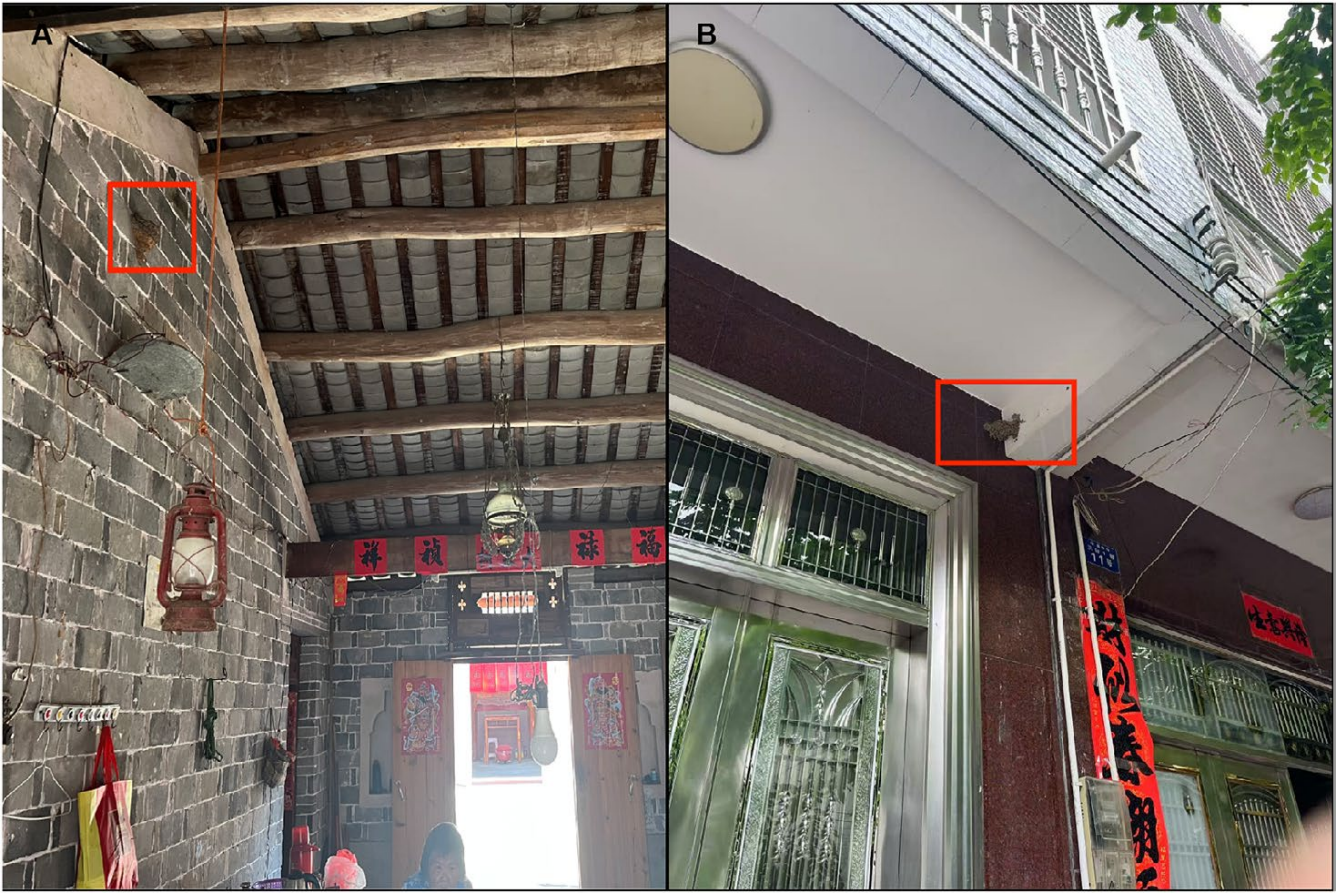
Building types at the study sites. (A) Traditional buildings with natural access points that facilitate barn swallow entry for indoor nesting. (B) Modern buildings with sealed doors and windows, restricting barn swallows to outdoor nesting only.

The predominance of outdoor nests contrasts with findings from temperate Denmark, where more than 99% of barn swallows nested indoors (Møller 2010). This discrepancy may partly reflect climatic differences, as warmer regions impose weaker thermal constraints on outdoor nesting, and Denmark harbors a depauperate predator community compared to southern China. However, we suggest that the pattern primarily reflects a passive response to building accessibility rather than an active preference. Traditional Chinese dwellings with wooden door and window frames provided abundant entry points, but the widespread adoption of glass doors and windows in modern construction has substantially reduced building permeability (Figure 7). Even where indoor access is possible, hygiene concerns and removal of nests by residents limit indoor nesting (Zeng et al. 2025). Consistent with this interpretation, the indoor nesting rate was highest in Heilongjiang (97.6%), where traditional architecture remains prevalent and residents actively facilitate swallow access (Video S1). Although our Heilongjiang sampling was limited to villages around Zhalong, barn swallows do breed in newer urban buildings elsewhere in the province. Overall, outdoor nesting appears to represent the outcome of a multifactor trade‐off involving building type, access, and resident attitudes, rather than an intrinsic preference. The increase in outdoor nests in Hainan over the past decade, hinted at by qualitative comparisons, coincides with rapid urbanization and architectural change.

### 4.4 Latitudinal Gradient in Outdoor Nesting

We found a marked latitudinal gradient in the proportion of outdoor nests (highest in tropical Hainan, lowest in temperate Heilongjiang). This pattern corresponds broadly to the climatic and architectural variation across our study sites—from the warm, urbanized landscape of Hainan to the cool, traditional rural setting of Heilongjiang. This pattern likely reflects the combined effects of climate, architecture, and cultural practices across the three study regions. Climatically, warmer regions impose weaker thermal constraints on outdoor nesting; during the breeding season, Hainan temperatures rarely fall below 25°C, while Heilongjiang's spring temperatures frequently approach freezing, favoring enclosed nest sites (Leung and Reid 2022). Architecturally, traditional dwellings in Heilongjiang have thick walls and resident‐made openings, whereas modern concrete buildings in Hainan with sealed exteriors limit indoor entry opportunities (Chen et al. 2025). Guizhou, with its mix of building types, occupies an intermediate position. Culturally, tolerating indoor nests and active facilitation are higher in Heilongjiang than in more urbanized and densely populated Hainan.

### 4.5 Height Differences Between Nest Types

Indoor nests were placed at significantly lower heights than outdoor nests. Because outdoor nests are more exposed to predators and brood parasites, higher placement may restrict access by larger birds (Nilsson 1984; Møller 1987; Liang et al. 2013), Indoors, predation and parasitism risks are lower. Moreover, barn swallows can distinguish household members from strangers (Liu et al. 2025); indoor spaces, occupied primarily by familiar individuals, may be perceived as safer, allowing lower nesting. We also observed that nests in inhabited buildings were placed higher than those in uninhabited buildings. Greater human disturbance could favor elevated placement (Cheng et al. 2023), but uninhabited buildings in our study were predominantly old structures with lower ceilings, potentially confounding the pattern. The small sample of nests in uninhabited buildings (*n* = 18) further limits this comparison.

Our study shows that barn swallows nested more frequently in inhabited buildings and in old structures, and that indoor nests were placed at lower heights. These findings are consistent with the human proximity hypothesis and reveal that outdoor nesting, contrary to prediction, is now predominant at lower latitudes, likely due to reduced building accessibility. Key avenues for future research include: (1) systematic measurements of building characteristics, predator assemblages, and microclimatic conditions to disentangle the drivers of geographic variation; (2) multivariate analyses to evaluate multiple predictors; (3) direct measurements of fitness outcomes and predation pressure to assess whether observed patterns reflect adaptive preferences; (4) broader geographic sampling to establish generality; and (5) comparable studies in other synanthropic species, such as the Eurasian tree sparrow ( *Passer montanus* ) and the red‐rumped swallow ( *Cecropis daurica* ). Although sampling was not fully synchronous across regions, we consider this a minor limitation given that all fieldwork was conducted during the peak breeding season and inter‐annual variation in nesting phenology is limited in this species.

## Author Contributions

**Cong Peng:** data curation (lead), formal analysis (lead), investigation (equal), methodology (equal), visualization (lead), writing – original draft (equal). **Kangning Luo:** investigation (equal), methodology (equal), resources (equal). **Kui Yan:** investigation (equal), methodology (equal), resources (equal). **Wei Liang:** conceptualization (lead), funding acquisition (lead), supervision (lead), validation (equal), writing – review and editing (equal).

## Funding

W.L. was supported by the National Key R & D Program of China (2023YFF1304600) and the specific research fund of the Innovation Platform for Academicians of Hainan Province. C.P. was funded by the Hainan Graduate Student Innovation Research Project (Qhyb2024‐161).

## Ethics Statement

The experiments comply with the current laws of China. No special permit was required for this study as it was not involved in animal or plant collection.

## Conflicts of Interest

The authors declare no conflicts of interest.

## Supporting information


**Video S1:** A case of high indoor breeding nests by barn swallows in a livestock‐keeping household in Heilongjiang, northeast China.

## Data Availability

Data used for this study and Video S1 are provided as Supporting Information—S1 and can be found at https://figshare.com/s/e5d9b2038bddde835329 (doi: 10.6084/m9.figshare.31746283).

## References

[ece374071-bib-0001] Banisaffar, M. , and A. A. Shabani . 2024. “Factors Influencing Nest Site Selection of the Laughing Dove ( *Spilopelia senegalensis* ) in an Urban Area in Karaj, Iran.” Ornis Hungarica 32: 117–137.

[ece374071-bib-0002] Barclay, R. M. R. 1988. “Variation in the Costs, Benefits, and Frequency of Nest Reuse by Barn Swallows ( *Hirundo rustica* ).” Auk 105: 53–60.

[ece374071-bib-0003] Bourgeois, K. , and É. Vidal . 2007. “Yelkouan Shearwater Nest‐Cavity Selection and Breeding Success.” Comptes Rendus Biologies 330: 205–214. 17434114 10.1016/j.crvi.2006.12.007

[ece374071-bib-0004] Campera, M. , J. Chavez , C. Humber , et al. 2024. “Impact of Cropland Management on Invertebrate Richness and Abundance in Agroforestry Systems in Bali, Indonesia.” Land 13: 493.

[ece374071-bib-0005] Chen, J. , N. Liu , C. Yan , and B. An . 2011. “Plasticity in Nest Site Selection of Black Redstart ( *Phoenicurus ochruros* ): A Response to Human Disturbance.” Journal of Ornithology 152: 603–608.

[ece374071-bib-0006] Chen, S. , Y. Liu , P. Li , et al. 2025. “Citizen Science Enabled Planning for Species Conservation in Urban Landscapes: The Case of Barn Swallows *Hirundo rustica* in Southern China.” Landscape Ecology 40: 65.

[ece374071-bib-0007] Cheng, L. , L. Zhou , C. Yu , Z. Wei , and C. Li . 2023. “Flexible Nest Site Selection of the Endangered Oriental Storks ( *Ciconia boyciana* ): Trade‐Off From Adaptive Strategies.” Avian Research 14: 100088.

[ece374071-bib-0008] Clarke, L. W. , G. D. Jenerette , and A. Davila . 2013. “The Luxury of Vegetation and the Legacy of Tree Biodiversity in Los Angeles, CA.” Landscape and Urban Planning 116: 48–59.

[ece374071-bib-0009] Cody, M. L. 1985. Habitat Selection in Birds. Academic Press.

[ece374071-bib-0010] Dor, R. , R. J. Safran , F. H. Sheldon , D. W. Winkler , and I. J. Lovette . 2010. “Phylogeny of the Genus *Hirundo* and the Barn Swallow Subspecies Complex.” Molecular Phylogenetics and Evolution 56: 409–418. 20152914 10.1016/j.ympev.2010.02.008

[ece374071-bib-0011] Feeney, W. E. , J. A. Welbergen , and N. E. Langmore . 2012. “The Frontline of Avian Brood Parasite–Host Coevolution.” Animal Behaviour 84: 3–12.

[ece374071-bib-0012] Guo, R. , Q. Wu , L. Chen , et al. 2023. “Nests Characteristics and Nest‐Site Selection of Common Cormorant in Longfeng Reserve, China.” Acta Ecologica Sinica 43: 2220–2227.

[ece374071-bib-0013] Hansell, M. H. 2000. Bird Nests and Construction Behaviour. Cambridge University Press.

[ece374071-bib-0014] Haynes, T. B. , J. A. Schmutz , M. S. Lindberg , and A. E. Rosenberger . 2014. “Risk of Predation and Weather Events Affect Nest Site Selection by Sympatric Pacific ( *Gavia pacifica* ) and Yellow‐Billed ( *Gavia adamsii* ) Loons in Arctic Habitats.” Waterbirds 37: 16–25.

[ece374071-bib-0015] Jeong, D. , Y. Shin , B. Lim , H. Serret , and Y. Jang . 2022. “Do Barn Swallows ( *Hirundo rustica gutturalis* ) Prefer to Breed in Inhabited Houses in South Korea?” Wilson Journal of Ornithology 134: 633–641.

[ece374071-bib-0016] Jokimäki, J. , J. Suhonen , T. Vuorisalo , L. Kövér , and M. L. Kaisanlahti‐Jokimäki . 2017. “Urbanization and Nest‐Site Selection of the Black‐Billed Magpie ( *Pica pica* ) Populations in Two Finnish Cities: From a Persecuted Species to an Urban Exploiter.” Landscape and Urban Planning 157: 577–585.

[ece374071-bib-0017] Jovani, R. , and J. L. Tella . 2004. “Age‐Related Environmental Sensitivity and Weather Mediated Nestling Mortality in White Storks *Ciconia ciconia* .” Ecography 27: 611–618.

[ece374071-bib-0018] Kesler, D. C. , and S. M. Haig . 2005. “Microclimate and Nest‐Site Selection in Micronesian Kingfishers.” Pacific Science 59: 499–508.

[ece374071-bib-0019] Kim, M. , O. S. Chung , and J. K. Lee . 2023. “The Relationship Between Nest Location Selection of Barn Swallows ( *Hirundo rustica* ) and Human Activity and Residence.” Scientific Reports 13: 23008. 38155232 10.1038/s41598-023-50149-6 PMC10754929

[ece374071-bib-0020] Leung, M. , and D. Reid . 2022. “Nesting Ecology of the Barn Swallow on Agricultural Lands in Yukon.” Western Birds 53: 309–326.

[ece374071-bib-0021] Li, X. , J. Bao , Y. Li , J. Wang , W. Yan , and W. Zhang . 2025. “Legacy and Luxury Effects: Dual Drivers of Tree Diversity Dynamics in Beijing's Urbanizing Residential Areas (2006–2021).” Forests 16: 1269.

[ece374071-bib-0022] Liang, W. , C. Yang , L. Wang , and A. P. Møller . 2013. “Avoiding Parasitism by Breeding Indoors: Cuckoo Parasitism of Hirundines and Rejection of Eggs.” Behavioral Ecology and Sociobiology 67: 913–918.

[ece374071-bib-0023] Liu, Y. , Y. Liu , and W. Liang . 2025. “Breeding Barn Swallows Recognize Householders From Strangers.” Animal Cognition 28: 33. 40299131 10.1007/s10071-025-01956-z PMC12041164

[ece374071-bib-0024] Ma, L. , C. Yang , J. Liu , J. Zhang , W. Liang , and A. P. Møller . 2018. “Costs of Breeding Far Away From Neighbors: Isolated Host Nests Are More Vulnerable to Cuckoo Parasitism.” Behavioural Processes 157: 327–332. 30059764 10.1016/j.beproc.2018.07.017

[ece374071-bib-0025] Maisey, A. C. , N. T. Carter , J. M. Incoll , and A. F. Bennett . 2016. “Environmental Influences on Variation in Nest‐Characteristics in a Long‐Term Study Population of the Superb Lyrebird, *Menura novaehollandiae* .” Emu ‐ Austral Ornithology 116: 445–451.

[ece374071-bib-0026] Martin, T. E. 1995. “Avian Life History Evolution in Relation to Nest Sites, Nest Predation, and Food.” Ecological Monographs 65: 101–127.

[ece374071-bib-0027] Millones, A. , and E. Frere . 2017. “How Nest Site Characteristics Influence Breeding Success in Red‐Legged Cormorants *Phalacrocorax gaimardi* .” Acta Ornithologica 52: 239–244.

[ece374071-bib-0028] Møller, A. P. 1987. “Intraspecific Nest Parasitism and Anti‐Parasite Behaviour in Swallows, *Hirundo rustica* .” Animal Behaviour 35: 247–254.

[ece374071-bib-0029] Møller, A. P. 1989. “Intraspecific Nest Parasitism in the Swallow *Hirundo rustica* : The Importance of Neighbors.” Behavioral Ecology and Sociobiology 25: 33–38.

[ece374071-bib-0030] Møller, A. P. 2010. “The Fitness Benefit of Association With Humans: Elevated Success of Birds Breeding Indoors.” Behavioral Ecology 21: 913–918.

[ece374071-bib-0031] Møller, A. P. , M. Díaz , and W. Liang . 2016. “Brood Parasitism and Proximity to Human Habitation.” Behavioral Ecology 27: 1314–1319.

[ece374071-bib-0032] Navarro, I. , M. Á. Farfán , J. E. Fa , and A.‐R. Muñoz . 2026. “Herbaceous Cover Management in Olive Groves Can Be Important for Aerial‐Feeding Birds. A Case Study in Southern Spain.” Journal for Nature Conservation 89: 127068.

[ece374071-bib-0033] Nilsson, S. G. 1984. “The Evolution of Nest‐Site Selection Among Hole‐Nesting Birds: The Importance of Nest Predation and Competition.” Ornis Scandinavica 15: 167–175.

[ece374071-bib-0034] Quintana, F. , and P. Yorio . 1998. “Competition for Nest Sites Between Kelp Gulls ( *Larus dominicanus* ) and Terns (*Sterna maxima* and *S. eurygnatha* ) in Patagonia.” Auk 115: 1068–1071.

[ece374071-bib-0035] Rae, S. , and D. Rae . 2014. “Orientation of Tawny Frogmouth ( *Podargus strigoides* ) Nests and Their Position on Branches Optimises Thermoregulation and Cryptic Concealment.” Australian Journal of Zoology 61: 469–474.

[ece374071-bib-0036] Rather, T. A. , S. Kumar , and J. A. Khan . 2020. “Multi‐Scale Habitat Modelling and Predicting Change in the Distribution of Tiger and Leopard Using Random Forest Algorithm.” Scientific Reports 10: 11473. 32651414 10.1038/s41598-020-68167-z PMC7351791

[ece374071-bib-0037] Ringhofer, M. , and T. Hasegawa . 2014. “Social Cues Are Preferred Over Resource Cues for Breeding‐Site Selection in Barn Swallows.” Journal of Ornithology 155: 531–538.

[ece374071-bib-0038] Safran, R. J. 2004. “Adaptive Site Selection Rules and Variation in Group Size of Barn Swallows: Individual Decisions Predict Population Patterns.” American Naturalist 164: 121–131. 10.1086/422198 15278838

[ece374071-bib-0039] Safran, R. J. 2006. “Nest‐Site Selection in the Barn Swallow, *Hirundo rustica* : What Predicts Seasonal Reproductive Success?” Canadian Journal of Zoology 84: 1533–1539.

[ece374071-bib-0040] Shields, W. M. 1984. “Factors Affecting Nest and Site Fidelity in Adirondack Barn Swallows ( *Hirundo rustica* ).” Auk 101: 780–789.

[ece374071-bib-0041] Teglhøj, P. G. 2018. “Artificial Nests for Barn Swallows *Hirundo rustica* : A Conservation Option for a Declining Passerine?” Bird Study 65: 385–395.

[ece374071-bib-0042] Tian, L. , Y. Liu , Y. Wu , Z. Feng , D. Hu , and Z. Zhang . 2024. “Migration Pattern of a Population of Barn Swallows ( *Hirundo rustica* ) Breeding in East Asian Tropical Region.” Avian Research 15: 100192.

[ece374071-bib-0043] Wang, J.‐S. , M.‐N. Tuanmu , and C.‐M. Hung . 2021. “Effects of Artificial Light at Night on the Nest‐Site Selection, Reproductive Success and Behavior of a Synanthropic Bird.” Environmental Pollution 288: 117805. 34351282 10.1016/j.envpol.2021.117805

[ece374071-bib-0044] Wang, Y. , S. Chen , P. Jiang , and P. Ding . 2008. “Black‐Billed Magpies ( *Pica pica* ) Adjust Nest Characteristics to Adapt to Urbanization in Hangzhou, China.” Canadian Journal of Zoology 86: 676–684.

[ece374071-bib-0045] Xu, Y. , Z. Cao , and B. Wang . 2020. “Effect of Urbanization Intensity on Nest‐Site Selection by Eurasian Magpies ( *Pica pica* ).” Urban Ecosystems 23: 1099–1105.

[ece374071-bib-0046] Yan, K. , and W. Liang . 2024. “Recognition and Rejection of Foreign Eggs of Different Colors in Barn Swallows.” Avian Research 15: 100202.

[ece374071-bib-0047] Yao, X. , Y. Cai , P. Ye , W. Liang , and C. Yang . 2023. “Nesting Preferences of Two Cavity‐Nesting Passerines in Human Houses.” Ornithological Science 22: 179–182.

[ece374071-bib-0048] Zeng, J. , Z. Luo , K. Guo , L. Tian , R. Zhang , and X. Zheng . 2025. “Barn Swallow ( *Hirundo rustica* ) Transfers Current‐Use Pesticides From Agricultural Fields to Houses.” Environmental Pollution 381: 126595. 40466998 10.1016/j.envpol.2025.126595

[ece374071-bib-0049] Zhang, J. , P. Santema , J. Li , W. Deng , and B. Kempenaers . 2023. “Brood Parasitism Risk Drives Birds to Breed Near Humans.” Current Biology 33: 1125–1129. 36805848 10.1016/j.cub.2023.01.047

[ece374071-bib-0050] Zhang, L. , H. Zhang , J. Wang , L. Zhang , Y. Cheng , and D. Wan . 2020. “Nest Site Selection and Breeding Success of Daurian Redstart *Phoenicurus auroreus* in Northeast China.” Acta Ecologica Sinica 40: 70–76.

[ece374071-bib-0051] Zhao, Y. , E. Pagani‐Núñez , Y. Liu , et al. 2022. “The Effect of Urbanization and Exposure to Multiple Environmental Factors on Life‐History Traits and Breeding Success of Barn Swallows ( *Hirundo rustica* ) Across China.” Avian Research 13: 100048.

[ece374071-bib-0052] Zhou, B. , J. Liu , and W. Liang . 2020. “Breeding in a Noisy World: Attraction to Urban Arterial Roads and Preference for Nest‐Sites by the Scaly‐Breasted Munia ( *Lonchura punctulata* ).” Global Ecology and Conservation 22: e00987.

